# Explore the Practice and Level of Knowledge of Otorhinolaryngology-Related Issues Among the Population of the Northern Borders Region in Saudi Arabia

**DOI:** 10.7759/cureus.51222

**Published:** 2023-12-28

**Authors:** Khalid M Alkhalifah, Mada Muteb Alanazi, Shahad I Almushayqih, Shaden M Aljurayyed, Nawal S Alanazi, Layan T Almazyad, Raghad Z Alenezi, Renad T Almazyad, Yahia Abdelgawad Elsayed Elboraei

**Affiliations:** 1 Unaizah College of Medicine and Medical Sciences, Qassim University, Unaizah, SAU; 2 College of Medicine, Northern Border University, Arar, SAU; 3 College of Medicine, Sulaiman Alrajhi University, Al Bukayriyah, SAU; 4 College of Medicine, Qassim University, Unaizah, SAU; 5 College of Applied Sciences, Northern Border University, Arar, SAU; 6 ENT Department, Faculty of Medicine, Northern Border University, Arar, SAU

**Keywords:** otolaryngology-head and neck surgeons, public awareness, knowledge, otorhinolaryngology, ent

## Abstract

Objective: The objective of this study was to evaluate public practice and the level of knowledge of common otorhinolaryngology-related issues among the population of the Northern Borders region of Saudi Arabia.

Methods: A descriptive cross-sectional questionnaire-based study was done in this research, employing data from 363 participants from the general population in the Northern Borders region of Saudi Arabia. The participants completed an online self-administered questionnaire and ensured anonymity. The questionnaire used in this study had been previously validated.

Results: Most of the respondents involved in this study were aged above 20 years (n = 326, 89.8%), and 248 had a medical background (68.3%). The study results show that 139 (38.2%) of the respondents had a good knowledge level, while 224 (61.8%) had poor knowledge about otorhinolaryngology-related issues. The results established a statistically significant association between the demographic information of age, gender, education level, and the level of knowledge about otorhinolaryngology-related issues with p-values < 0.05 (0.001, 0.003, and 0.002), respectively. There were no statistically significant association between marital status, place of residence, occupation, medical background, and the level of knowledge about otorhinolaryngology-related issues (with p > 0.05).

Conclusion: The study found that less than half of the participants had good knowledge of otorhinolaryngology; elder and female participants showed better understanding. Sociodemographic factors, such as age, gender, and education, were statistically linked to knowledge levels. The findings highlight a need for increased public awareness efforts by the medical community regarding otorhinolaryngology issues in Saudi Arabia.

## Introduction

Ear, nose, and throat (ENT) disorders refer to a group of diseases that affect the ear, nose, and throat. Moreover, it is referred to as otolaryngology [1]. They are exceedingly common, can create severe disturbance in the lives of sufferers, and are frequently treated in an emergency department (ED) [2]. Furthermore, ENT issues are among the most common reasons why people contact their primary care physicians [3-4].

Otolaryngologists treat a wide variety of disorders, including upper respiratory tract infections, allergic rhinitis (AR), otitis media, and potentially fatal ailments, including nasopharyngeal cancer. Another key fact to remember is that there are two types of ENT diseases, which are congenital and acquired ENT disorders. To give an illustration of these two types, the congenital type is a condition or trait that exists at birth, while the acquired type may be caused by trauma, pathogenic organisms, or inflammatory processes [5].

For instance, AR is one of the most prevalent disorders recognized by otolaryngologists [6], with a frequency ranging from 10% to 30% in adults and an even higher prevalence in children [7-8]. In addition, the cost of AR is predicted to range from $1.6 to 4.9 billion with a total cost of $9.7 billion in the United States alone. Therefore, ENT problems are substantial sources of morbidity and place a significant strain on healthcare systems [9].

Previous research worldwide suggests that awareness of and attitudes concerning ENT-related disorders are insufficient [10-13]. There is a published research entitled “Public Perception in the Management and Prevention of Common Ear, Nose, and Throat Diseases in Saudi Arabia,” which aimed to evaluate the awareness and knowledge of the attitudes toward common ENT-related diseases among Saudi Arabian adults in Riyadh. As a result, only 2.3% of the individuals had great knowledge, while 18.4% had good knowledge. The remaining majority (79.4%) had a limited awareness of ENT-related disorders (<50% score). Thus, people in Riyadh had a general lack of knowledge of frequent ENT-related disorders. As a result, these findings emphasize the need to raise awareness through educational initiatives, particularly community-based public health campaigns, as the participants in this study recognized the community as a primary information source. Moreover, these public awareness campaigns can help to reduce the occurrence and spread of contagious ENT disorders [14]. 

Furthermore, this research leads us to do a study that aims to evaluate public practice and the level of knowledge of common otorhinolaryngology-related issues among the population of the Northern Borders region of Saudi Arabia.

## Materials and methods

Study design, duration, and setting

The study is a descriptive cross-sectional questionnaire-based study. A cross-sectional survey was conducted among the general population in the Northern Borders region of Saudi Arabia from October 11 to November 11, 2023. 

Sample size and sampling technique

Using Epi Info software version 7.2.2.6 (Centers for Disease Control and Prevention, Atlanta, GA, USA), the sample size was computed with a 50% expected frequency, a 95% confidence interval, and 80% power. The estimated size of the sample was 384. The recruitment of participants was conducted using a convenient sampling technique, and their informed consent was duly requested. The objectives of the research were communicated to the participants who subsequently provided informed consent.

Inclusion criteria

Northern Borders citizens aged 18 and older, proficient in Arabic or English, and willing to participate are required to complete the questionnaire.

Data collection tool

Data were collected through an online self-administered questionnaire that was used in a previous study [14], which was distributed to the participants after they receive a complete explanation of the purpose of this study. To assess the level of public awareness regarding the prevention and management of prevalent otorhinolaryngology-related diseases, the authors devised a customary survey comprising 23 items organized into three primary sections: the first section was for demographic information (age, gender, marital status, level of education, and occupation), the second section was for general knowledge and addressed in eight inquiries, and the third section was for ear and hearing diseases and nose, throat, and laryngeal diseases. A survey comprising an inquiry regarding the respondents' place of residence and exclusively those residing in the Northern Borders region in Saudi Arabia was incorporated. After the study was carried out and a pilot questionnaire was developed, the questionnaire underwent revisions. The knowledge inquiries incorporated a self-reported Likert scale consisting of the following responses: "true," "false," or "I don't know." An attempt was made by a board-certified otolaryngologist to dispel the most prevalent misunderstanding among the Saudi populace and to address the most prevalent otorhinolaryngology-related conditions.

Data analysis

The extraction process included revising, coding, and inputting the data into IBM SPSS Statistics for Windows, version 22 (released 2013; IBM Corp., Armonk, New York, United States). Two-tailed tests were utilized in all statistical analyses, with consideration given to a P-value below 0.05 as statistically significant. Regarding knowledge and awareness, one point was deducted for each accurate response, and the cumulative score of individual scores for various items was then computed.

Ethical considerations

The research was conducted after obtaining the required approvals from the Regional Committee for Research Ethics, Northern Border University, Arar, Saudi Arabia (no. 87/44/H).

## Results

As shown in Table 1, a total of 363 participants completed the online questionnaire. The vast majority (n = 326, 89.8%) of the participants were more than 20 years old, with a mean and standard deviation of 34.14 ± 13.79 years. The majority of them (n = 279, 76.9%) were females. More than half (n = 195, 53.7%) of the participants were single. It was evident that the majority (n = 228, 62.8%) resided in Arar. Most participants (n = 269, 74.1%) had attained a university education level. More than half (n = 248, 68.3%) had a medical background.

**Table 1:** Sociodemographic information of the participants (N = 363) Sociodemographic information presented in frequencies (n) and proportion (%).

Sociodemographic information	Category	Frequency and proportion n (%)
Age	Less than 20 years	37 (10.2)
	More than 20 years	326 (89.8)
Gender	Male	84 (23.1)
	Female	279 (76.9)
Marital status	Single	195 (53.7)
	Married	150 (41.3)
	Widowed	5 (1.4)
	Divorced	13 (3.6)
Place of residence	Al-Awiqila	7 (1.9)
	Arar	228 (62.8)
	Qurayyat	16 (4.4)
	Rafha	79 (21.8)
	Sakaka	4 (1.1)
	Turaif	29 (8.0)
Educational level	Less than high school	9 (2.5)
	Secondary	60 (16.5)
	Diploma	5 (1.4)
	University	269 (74.1)
	Post-graduate	20 (5.5)
Occupation	I work in other sectors	122 (33.6)
	I work in the medical sector	22 (6.1)
	Student in other sectors	62 (17.1)
	Student in the medical sector	75 (20.7)
	Unemployed	82 (22.6)
Medical background	Yes	248 (68.3)
	No	115 (31.7)

Table 2 depicts the knowledge about the common otorhinolaryngology-related issues of the participants. The vast majority (n = 254, 70.0%) of the participants provided correct answers on the importance of test hearing in newborns; 286 (78.9%) were aware that it is recommended to get the flu vaccines annually; more than half (n = 233, 64.2%) provided the correct answers on the importance of using vitamin C to treat and prevent common cold. Notably, more than 50% of the respondents were not sure of the importance of influenza vaccine for prevention in patients with comorbidities. In addition, the vast majority of the participants (n = 277, 76.3%) were not aware of the use of olive oil as an ear drop to treat ear diseases, and the majority (n = 228, 62.8%) were not aware of the definition of vertigo.

**Table 2:** General knowledge about common otorhinolaryngology-related issues (presented in frequencies (n) and proportion (%)).

Questions	Categories	Frequency and proportion n (%)
Cotton swabs are considered a safe way to clean the ear.	Correct	93 (25.6)
	Wrong	237 (65.3)
	I don’t know	33 (9.1)
Newborns’ hearing can be screened.	Correct	254 (70.0)
	Wrong	30 (8.3)
	I don’t know	79 (21.8)
Are antibiotics a treatment for viral upper respiratory infections?	Correct	151 (41.6)
	Wrong	115 (31.7)
	I don’t know	97 (26.7)
It is recommended to get the flu vaccine annually?	Correct	286 (78.9)
	Wrong	36 (9.9)
	I don’t know	41 (11.3)
Influenza vaccine is not recommended for patients with high blood pressure and high blood sugar levels.	Correct	86 (23.7)
	Wrong	132 (36.4)
	I don’t know	145 (39.9)
Vitamin C can prevent and treat the common cold.	Correct	233 (64.2)
	Wrong	85 (23.4)
	I don’t know	45 (12.4)
Can using olive oil as ear drops treat ear diseases?	Correct	86 (23.7)
	Wrong	191 (52.6)
	I don’t know	86 (23.7)
Vertigo and dizziness are the same thing.	Correct	95 (26.2)
	Wrong	228 (62.8)
	I don’t know	40 (11.0)

Table 3 depicts the knowledge about ear and hearing diseases among participants. The vast majority (n = 326, 89.8%) of the participants provided correct answers on the effects of hearing loss on school performance among children; 298 (82.1%) were correct on the effect of hearing loss on social life; more than half (n = 249, 68.6%) of the participants were aware that constant exposure to noise can cause hearing loss; 302 (83.2%) of the participants had correct knowledge on the causes of vertigo; and more than half (n = 198, 54.5%) knew that every ear pain does not necessarily mean that there is a middle ear infection. The vast majority (n = 316, 87.1%) were aware that sudden hearing loss is an emergency condition that requires immediate medical intervention. However, a majority of the participants (n = 296, 74.4%) were not sure whether the hearing aid could be used for children under 12 months.

**Table 3:** Knowledge about ear and hearing diseases (presented in frequencies (n) and proportion (%)).

Questions	Categories	Frequency and proportion n (%)
Can hearing loss in children cause distraction and affect school performance?	Correct	326 (89.8)
	Wrong	12 (3.3)
	I don’t know	25 (6.9)
Can hearing loss affect social life?	Correct	298 (82.1)
	Wrong	40 (11.0)
	I don’t know	25 (6.9)
Constant exposure to noise can damage your hearing and may cause hearing loss.	Correct	249 (68.6)
	Wrong	42 (11.6)
	I don’t know	72 (19.8)
Hearing aids are one of the most common ways to improve hearing in elderly patients.	Correct	282 (77.7)
	Wrong	20 (5.5)
	I don’t know	61 (16.8)
Some inner ear infections can cause vertigo.	Correct	302 (83.2)
	Wrong	13 (3.6)
	I don’t know	48 (13.2)
Hearing aids can be used for children under 12 months.	Correct	94 (25.6)
	Wrong	85 (23.4)
	I don’t know	184 (50.7)
Every ear pain necessarily means that there is a middle ear infection.	Correct	71 (19.6)
	Wrong	198 (54.5)
	I don’t know	94 (25.9)
Sudden hearing loss is an emergency and requires immediate medical evaluation.	Correct	316 (87.1)
	Wrong	7 (1.9)
	I don’t know	40 (11.0)

Table 4 shows the knowledge about the nose, throat, and laryngeal diseases among participants. The majority of the participants (n = 190, 52.3%) provided correct answers on best ways of handling nosebleeds and the safety use of congestion relief drops (n = 208, 57.3%). In addition, more than half of the participants (n = 241, 63.4%) had knowledge that voice abuse causes voice cord abuse, while the vast majority (n = 306, 84.3%) of the respondents were aware that smoking can cause throat cancer.

**Table 4:** Knowledge about nose, throat, and laryngeal diseases (presented in frequencies (n) and proportion (%)).

Knowledge about problems	Categories	Frequency and proportion n (%)
The best way to deal with bleeding is to tilt the head back.	Correct	111 (30.6)
	Wrong	190 (52.3)
	I don’t know	62 (17.1)
Congestion relief drops are safe for long-term use.	Correct	56 (15.4)
	Wrong	208 (57.3)
	I don’t know	99 (27.2)
The first step to controlling nasal allergy symptoms (allergic runny nose) is to stay away from the specific irritants that cause the symptoms.	Correct	310 (85.4)
	Wrong	13 (3.6)
	I don’t know	40 (11.0)
Tonsillectomy procedure causes obesity.	Correct	67 (18.5)
	Wrong	160 (44.1)
	I don’t know	136 (37.5)
Tonsillectomy procedure can lead to immunodeficiency.	Correct	148 (40.8)
	Wrong	110 (30.3)
	I don’t know	105 (29.0)
Excessive use of the voice can cause vocal cord disorders.	Correct	241 (63.4)
	Wrong	58 (16.0)
	I don’t know	64 (17.6)
Smoking can cause throat cancer.	Correct	306 (84.3)
	Wrong	10 (2.8)
	I don’t know	47 (12.9)

Table 5 presents the relationship between participants’ sociodemographic information and level of knowledge about otorhinolaryngology-related issues. The results established statistically significant association between age, gender, and education level with p-values < 0.05 (0.001, 0.003, and 0.002, respectively) and the level of knowledge about otorhinolaryngology-related issues. There were no statistically significant association between marital status, place of residence, occupation, medical background, and the level of knowledge about otorhinolaryngology-related issues (p > 0.05). The study results show that 139 (38.2%) had a good knowledge level, while 224 (61.8%) had poor knowledge about otorhinolaryngology-related issues.

**Table 5:** Association between sociodemographic information and level of knowledge about otorhinolaryngology-related issues. * significant at p < 0.05 level.

	Level of knowledge			
Variables	Category	Poor	Good	p-value
Age	Less than 20 years	23 (62.2)	14 (37.8)	0.001*
	More than 20 years	170 (52.1)	156 (47.9)	
Gender	Male	56 (66.7)	28 (33.3)	0.003*
	Female	175 (62.9)	104 (37.1)	
Marital status	Single	127 (64.9)	68 (35.1)	0.156
	Married	91 (60.6)	59 (39.4)	
	Widowed	3 (68.3)	2 (31.7)	
	divorced	9 (66.5)	4 (33.5)	
Place of residence	Al-Awiqila	4 (63.9)	3 (36.1)	0.205
	Arar	137 (59.9)	91 (40.1)	
	Qurayyat	10 (64.5)	6 (35.5)	
	Rafha	53 (67.6)	26 (32.4)	
	Sakaka	3 (66.1)	1 (33.9)	
	Turaif	19 (65.3)	10 (34.7)	
Education level	Less than high school	6 (63.9)	3 (36.1)	0.002*
	Secondary	41 (67.6)	19 (32.4)	
	Diploma	3 (61.6)	2 (38.4)	
	University	162 (60.1)	107 (39.9)	
	Post-graduate	12 (58.8)	8 (41.2)	
Occupation	I work in other sectors	84 (68.7)	38 (31.3)	0.269
	I work in the medical sector	7 (31.8)	15 (68.2)	
	Student in other sectors	38 (61.5)	24 (38.5)	
	Student in the medical sectors	43 (57.6)	32 (42.4)	
	Unemployed	55 (67.4)	27 (32.6)	
Medical background	Yes	135 (54.3)	113 (45.7)	0.114
	No	71 (62.1)	44 (37.9)	

## Discussion

The study aimed to evaluate the public practice and the level of knowledge of common otorhinolaryngology-related issues among the population of the Northern Borders region of Saudi Arabia. The sample for the current study primarily consisted of participants aged more than 20 years, with a predominance of females, majority of them were singles, were residents of Arar with university education, worked in other fields (not medical field), and had a medical background. 

The findings revealed that only 38.2% (n = 139) of the participants had good knowledge about otorhinolaryngology-related issues. The study showed that the participants older than 20 years had better knowledge of otorhinolaryngology-related problems than those younger than 20 years. It was revealed that the female participants showed a higher level of knowledge about otorhinolaryngology-related issues. The findings of the study mirror the findings of the study conducted by Alassaf et al., which revealed that women had higher knowledge regarding common problems of the ear, nose, and throat due to the fact that women are ordinarily considered as the caretakers of family health matters rather than males [15]. Most of the participants showed good knowledge regarding the importance of test hearing in newborns (n = 254, 70.0%), and 78.9% (n = 286) of the participants had good knowledge about the importance of annual vaccination as a way of preventing flu. More than half (n = 233, 64.2%) of the participants had good knowledge of the use of vitamin C in the treatment and prevention of the common cold. The current findings are consistent with the findings of a study conducted by Jalaladdin et al. in Saudi Arabia, which established a good awareness regarding the importance of using vaccines and vitamin C in preventing influenza [16].

The study noted that the majority of the participants had good knowledge regarding the effects of hearing loss on children's school performance (n = 326, 89.8%) and their social life (n = 298, 82.1%). In addition, the study noted that more than half of the participants had good knowledge of the effects of constant exposure to noise (n = 249, 68.6%) and the causes of vertigo (n = 302, 83.2%). The findings concur with those of the study conducted by Almutairi et al., which revealed good knowledge on the cause vertigo and the common hearing loss among children among the general population in Saudi Arabia [17].

The findings revealed that majority of the participants had good knowledge on the appropriate measures of handling nosebleed (n = 190, 52.3%), safe use of congestion relief drops (n = 208, 57.3%), and ways of controlling nasal allergy symptoms (n = 310, 85.4%). The vast majority (n = 316, 87.1%) of the respondents had knowledge that sudden hearing loss is an emergency condition that requires immediate medical intervention. The findings are consistent with those of Alshehri et al., which reported a substantial level of knowledge on measures of handling epistaxis and the appropriate action to be taken in case of a medical emergency regarding otorhinolaryngology-related issues [18]. 

The results of this study may have been predisposed to some limitations. A descriptive cross-sectional questionnaire-based study design of this nature has a limitation attributed to its inability to assess causal relationships. In addition, considering that the study involved the administration of online questionnaires, the study relied on respondents accurately documenting their response without the ability to check this, which may have contributed to a potential bias. Moreover, the study findings cannot be generalized to the entire Saudi Arabia population given that it was conducted in only one region, i.e., Northern Borders region.

## Conclusions

The study revealed that 38.2% of the participants had good knowledge about otorhinolaryngology-related issues. The participants older than 20 years had better knowledge of common otorhinolaryngology problems than those younger than 20 years. Female participants showed a higher level of knowledge of problems related with otorhinolaryngology. The study established a statistically significant association between sociodemographic information of age, gender, education level, and the level of knowledge about otorhinolaryngology-related issues. There is a need for concerted efforts by the medical community to increase public awareness about otorhinolaryngology problems among the general population in Saudi Arabia.

## References

[1] (20223). Definition of ENT. https://www.merriam-webster.com/dictionary/ENT.

[2] Raj A, Wadhwa V, Jain A (2018). Epidemiological profile of ENT emergencies: our experience. Indian J Otolaryngol Head Neck Surg.

[3] McCormick A, Fleming D, Charlton J (1995). Morbidity statistics from general practice. 4th National Study 1992-1993.

[4] Finley CR, Chan DS, Garrison S (2018). What are the most common conditions in primary care? Systematic review. Can Fam Physician.

[5] Zeeshan M, Zeb J, Saleem M (2018). ENT diseases presenting to a tertiary care hospital. Endocrinol Metab.

[6] Pillsbury HC 3rd, Cannon CR, Sedory Holzer SE (2000). The workforce in otolaryngology-head and neck surgery: moving into the next millennium. Otolaryngol Head Neck Surg.

[7] Dykewicz MS, Fineman S, Skoner DP, Nicklas R, Lee R, Blessing-Moore J (1998). Diagnosis and management of rhinitis: complete guidelines of the joint task force on practice parameters in allergy, asthma and immunology. Ann Allergy Asthma Immunol.

[8] Wright AL, Holberg CJ, Martinez FD, Halonen M, Morgan W, Taussig LM (1994). Epidemiology of physician-diagnosed allergic rhinitis in childhood. Pediatrics.

[9] Reed SD, Lee TA, McCrory DC (2004). The economic burden of allergic rhinitis: a critical evaluation of the literature. Pharmacoeconomics.

[10] Luryi AL, Yarbrough WG, Niccolai LM (2014). Public awareness of head and neck cancers: a cross-sectional survey. JAMA Otolaryngol Head Neck Surg.

[11] Crandell C, Mills TL, Gauthier R (2004). Knowledge, behaviors, and attitudes about hearing loss and hearing protection among racial/ethnically diverse young adults. J Natl Med Assoc.

[12] Joubert K, Sebothoma B, Kgare KS (2017). Public awareness of audiology, hearing and hearing health in the Limpopo Province, South Africa. S Afr J Commun Disord.

[13] Knobel KA, Lima MC (2012). Knowledge, habits, preferences, and protective behavior in relation to loud sound exposures among Brazilian children. Int J Audiol.

[14] Alkholaiwi F, Alharbi MM, Aldayel AY, Shadid AM, Almutairi FE, Dahmash AB (2020). Public perception in the management and prevention of common ear, nose, and throat diseases in Saudi Arabia. J Nat Sci Med.

[15] Alassaf HI, ALturki AY, Almansour NM (2023). Basic ear, nose, and throat knowledge among medical students: a multi-institutional study reflecting the current stage of ear, nose, and throat curriculum in undergraduate medical education. J Nat Sci Med.

[16] Jalaladdin MS, Sindi EE, Kabli AF (2023). An assessment of the knowledge and awareness of common otorhinolaryngology-related issues among school and university students in Makkah City, Saudi Arabia: a cross-sectional study. Cureus.

[17] Almutairi AN, Altuaysi AM, Alwhaid MS (2022). Knowledge and attitude of the general population regarding infant hearing loss in Saudi Arabia. J Family Med Prim Care.

[18] Alshehri KA, Alqulayti WM, Yaghmoor BE, Alem H (2019). Public awareness of ear health and hearing loss in Jeddah, Saudi Arabia. S Afr J Commun Disord.

